# Valorization of Rice Straw via Hydrotropic Lignin Extraction and Its Characterization

**DOI:** 10.3390/molecules26144123

**Published:** 2021-07-06

**Authors:** Chongxin Yin, Min Wang, Qingzhi Ma, Huiyang Bian, Hao Ren, Hongqi Dai, Jinlan Cheng

**Affiliations:** 1 Jiangsu Co-Innovation Center for Efficient Processing and Utilization of Forest Resources, Jiangsu Provincial Key Lab of Pulp & Paper Science & Technology, Nanjing Forestry University, Nanjing 210037, China; yinchongxin1115@163.com (C.Y.); miminwang@126.com (M.W.); hybian1992@njfu.edu.cn (H.B.); renhao@njfu.edu.cn (H.R.); daihq@njfu.com.cn (H.D.); 2 Zhejiang Provincial Collaborative Innovation Center of Agricultural Biological Resources Biochemical Manufacturing, Zhejiang University of Science and Technology, Hangzhou 310023, China; maqingzhi@zust.edu.cn

**Keywords:** *p*-Toluene sulfonic acid, lignin, rice straw, high valorization

## Abstract

Rice straw hydrotropic lignin was extracted from *p*-Toluene sulfonic acid (*p*-TsOH) fractionation with a different combined delignification factor (CDF). Hydrotropic lignin characterization was systematically investigated, and alkaline lignin was also studied for the contrast. Results showed that the hydrotropic rice straw lignin particle was in nanometer scopes. Compared with alkaline lignin, the hydrotropic lignin had greater molecular weight. NMR analysis showed that β-aryl ether linkage was well preserved at low severities, and the unsaturation in the side chain of hydrotropic lignin was high. H units and G units were preferentially degraded and subsequently condensed at high severity. High severity also resulted in the cleavage of part β-aryl ether linkage. ^31^P-NMR showed the decrease in aliphatic hydroxyl groups and the increasing carboxyl group content at high severity. The maximum weight loss temperature of the hydrotropic lignin was in the range of 330–350 °C, higher than the alkaline lignin, and the glass conversion temperature (T_g_) of the hydrotropic lignin was in the range of 107–125 °C, lower than that of the alkaline lignin. The hydrotropic lignin has high β-aryl ether linkage content, high activity, nanoscale particle size, and low T_g_, which is beneficial for its further valorization.

## 1. Introduction

Rice straw is a byproduct of rice production and one of the most abundant, renewable, and low-cost agricultural wastes in the world. The general method that enterprises have adopted is burning, but it reduces the utilization value of lignin and causes some pollution to the environment [1,2]. Effective fractionation and full utilization of straw components can reduce environmental pollution and resource consumption [3,4,5]. Lignocellulosic biomass is primarily comprised of cellulose, hemicellulose, and lignin components. The lignin component is the second greatest inexhaustible natural organic polymer, and it represents more than 20% of the total mass of the Earth’s biosphere [6]. The high valorization of lignin into the production of value-added chemicals is now recognized as a real challenge in terms of both sustainability and environmental protection. In a typical biorefinery, linocellulose is first fractioned by pretreatment, which is energy intensive, and predominantly focuses on producing high-quality cellulose. Many fractionation processes have been developed, such as alkaline, dilute acid, SPORL, organosolv, and SO_2_-catalyzed steam explosion, deep eutectic solvent, and other pretreatments [7,8,9,10,11]. Under these processing conditions the lignin is structurally modified, rending its further catalytic valorization very challenging [12]. Therefore, hydrotropic fraction is of great significance for finding a kind of pretreatment method that has relatively mild processing conditions and is beneficial for utilizing all components of the lignocellulose.

Most works have focused on alkaline and kraft lignin [13,14], and hydrotropic lignin has only received attention in recent years. Hydrotropes are a class of amphiphilic molecules that cannot form well-organized structures, such as micelles, in water but do increase the aqueous solubility of organic molecules [15]. Hydrotropes increase the solubility of organic molecules in water by mediating the interactions between hydrophilic and hydrophobic molecules. p-tert-butylbenzenesulfonate, sodium cumenesulfonate, sodium *p*-toluenesulfonate, and 3,4-dimethylbenzenesulfonate are some commen hrdrotopes. *p*-Toluene sulfonic acid (*p*-TsOH) is an efficient acid hydrotrope that can increase the solubility of hydrophobic substance lignin in the water phase [16,17]. *p*-TsOH hydrotropic fractionation is extensively studied due to environmentally friendly in terms of the ease separation, recovery and energy-efficient. 90% Lignin can be removed at 80 °C within 20 min at 80 wt% of *p*-TsOH, and the effect is equivalent to using a basic aqueous solvent to react for more than 10 h at a temperature of ≥150 °C or an alkaline method for 2 h at 150 °C [18]. The ability of hydrotropes to increase the solubility of organics in water is often strongest when the hydrotrope concentration is sufficient to induce the formation of associated structures. The concentration at which self-association begins is denoted as the minimum hydrotrope concentration (MHC). By diluting the spent liquor of *p*-TsOH fraction with water to below the MHC (12.5%), lignin can be precipitated [19,20]. The lignin obtained under mild reaction conditions has a light color, a low glass transition temperature, and a high β-aryl ether bond content [21]. When using a flow reaction instead of a batch reaction process, the results showed that the yield of lignin was as high as 80% [22]. *p*-TsOH can be reused after the steps of lignin precipitation, reconcentration, and dehydration of xylose into furfural. The *p*-TsOH after the reaction does not lose activity [23]. The low-temperature fractionation process could substantially reduce capital and operating costs for applications [24]. The characterization of rice straw hydrotropic lignin extracted from *p*-TsOH was studied and compared with alkaline lignin. This study provides a theoretical basis for the high value-added utilization of rice straw.

## 2. Results and Discussion

### 2.1. p-TsOH Fractionated Rice Straw Lignin

**Figure 1:**
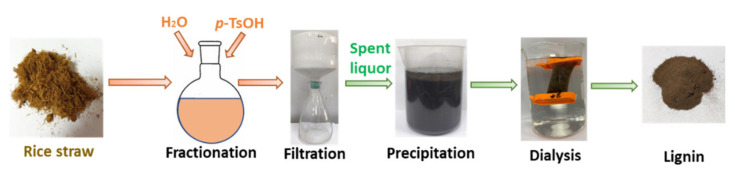
Experimental schematic flow diagram.

**Figure 2:**
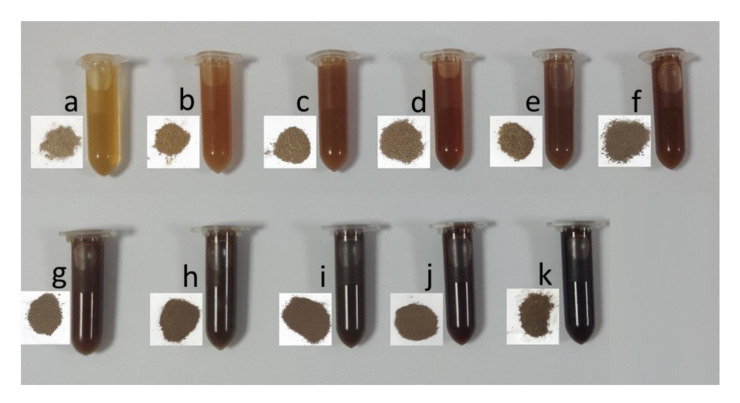
Pictures of *p*-TsOH fractionated rice straw lignin and the spent liquor (in the bottles). (**a**) P20T70t60; (**b**) P45T60t45; (**c**) P30T70t45; (**d**) P60T60t30; (**e**) P30T80t30; (**f**) P45T70t30; (**g**) P60T70t15; (**h**) P45T80t15; (**i**) P45T70t60; (**j**) P45T80t60; (**k**) P60T80t45.

A *p*-Toluene sulfonic acid (*p*-TsOH) fractionation schematic is shown in Figure 1. Rice straw lignin obtained under the different conditions with *p*-TsOH concentration and temperature is shown in Figure 2. PxxTyytzz stands for fractionation under *p*-TsOH concentration at xx wt% and yy °C for zz min. Reaction severity-based kinetic analysis was effective in predicting lignin dissolution in hydrotropic fractionation wood fractionation [21]. Studies have shown that acid pretreatment such as SPORL and *p*-TsOH fractionation can be divided into two stages: fast reaction and slow reaction [25,26]. The combined delignification factor (CDF) can be used to evaluate and predict the lignin removal rate [27]. The CDF can be obtained by the Equations (1) and (2). Where C is the molar concentration of *p*-TsOH (mol/L), R is the universal gas constant, 8.314 (J/(mol·K)), t is the reaction time (min), T is the reaction temperature (K), and E is the activation energy (J/mol), α and β are fitting parameters, θ′ is the component of slow-reacting lignin, and f′ is the ratio of the reaction rate between slow to fast lignin. θ′_R_ is the residual lignin. L_R_ is the fractions of residual lignin in solids.

(1) $$L_{R}=\left(1-\;\theta \prime -\theta_{R}\prime \right)e^{-\mathrm{CDF}}+\;\theta \prime \cdot e^{-\;f\prime \cdot \mathrm{CDF}}+\theta_{R}\prime$$

(2) $$\mathrm{CDF}=[\exp(\alpha \prime -\frac{E\prime }{\mathrm{RT}}+\beta \prime C)]\cdot C\cdot t$$

**Figure 3:**
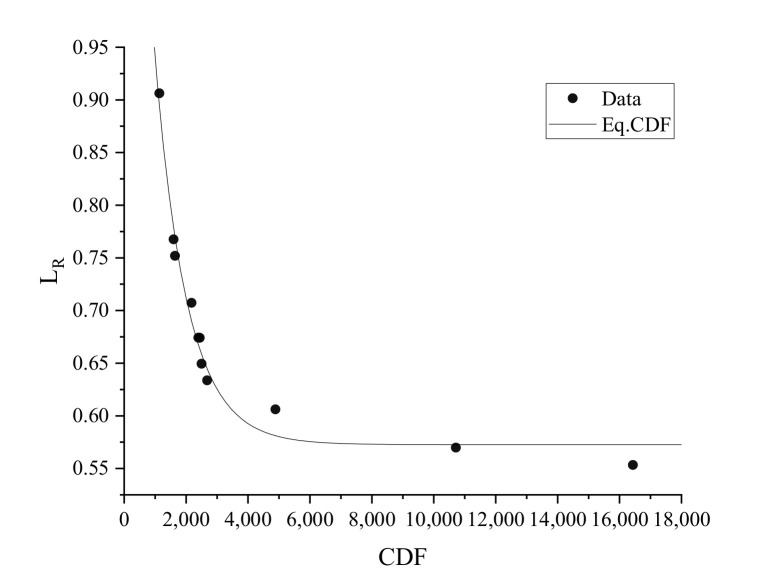
CDF curve of *p*-TsOH fractionation with rice straw.

The CDF curve was obtained according to the degree of lignin removal in *p*-TsOH fractionated rice straw. The high value of CDF indicates high severity. Elevated fractionation temperature and ex-tended fractionation time resulted in more lignin removal. The lignin removal rate and CDF curves are presented in Table 1 and Figure 3. The CDF at the inflection point of the curve is about 4000.

### 2.2. AFM Analysis

**Figure 4:**
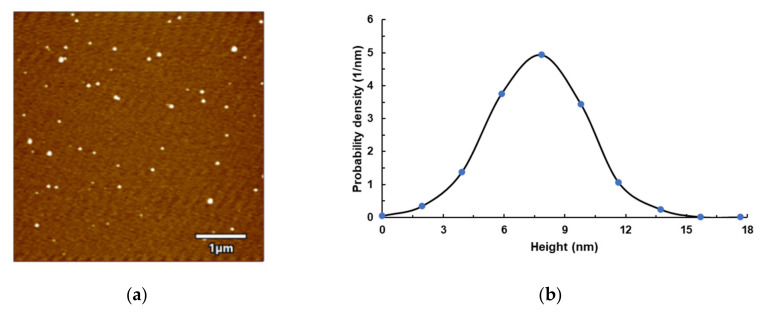
AFM images of lignin and height probability distribution profile: (**a**) P45T80t60 lignin image; (**b**) lignin height distribution profile.

AFM images and AFM topography of P45T70T60 (around the CDF curve inflection point) lignin sample are shown in Figure 4. The mean height of lignin was about 8 nm, and it was spherical. Nano lignin has a larger specific surface area and has great application value in the field of biomass functional materials. Recently, the development of nanoparticles (NPs) from lignin has gained interest due to its advantageous nature for drug delivery systems, delivery of hydrophobic molecules, improvement of the UV barrier, and as a reinforcing agent in nanocomposites, and its antibacterial and antioxidant applications. NPs developed from lignin have also been used as an alternative to inorganic NPs due to some safety issues raised in recent years [28,29,30].

### 2.3. Particle Size Distribution of Lignin

**Figure 5:**
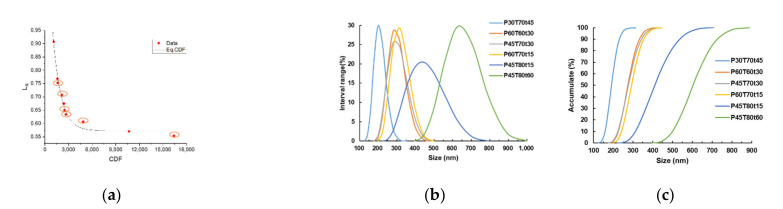
Size measurements of lignin: (**a**) The position of lignin obtained by *p*-TsOH fractionation on the CDF curve; (**b**) Distribution diagram of lignin particle size interval; (**c**) The cumulative distribution diagram of lignin particle size.

Hydrotropic lignin samples were chosen according to the CDF curve, which is circled in Figure 5a, including mild, medium, and severe severity. Average particle size of these lignin samples is ranged from 140 to 792 nm. Fractionation severity affects lignin particle size. When the conditions were severe, the average particle size of lignin increased, and the particle dispersity also increased. The lignin particle is easy to flocculate in water, and the average particle size of lignin detected may not contribute to the single particle, but the lignin aggregate.

### 2.4. Gel Permeation Chromatographic (GPC) Analyses

The weight-average (Mw) and number-weight (Mn) molecular weights and polydispersity index (PDI) of the hydrotropic lignin and the alkaline lignin are shown in Table 2. The hydrotropic lignin had greater molecular weight, the Mw of hydrotropic lignin was all over 16,055 Da, the Mn was all over 2400 Da, and the PDI of hydrotropic lignin was greater than 3.9. The Mw of alkaline lignin was only 4397 Da, the Mn was 1428 Da, and the PDI was 3.08. During the reaction process, NaOH can break the ether bond between lignin molecules, so the lignin macromolecules degraded, and the molecular weight decreased. At low fractionation severity such as P30T80t30, with Mw = 16055 Da and PDI = 5.58, the Mw of hydrotropic lignin decreased a little because of the partial degradation. The molecular weight decreased with the increased severity under fast reaction stages in the CDF curve. But at high severity in a slow reaction phase such as P60T80t45, the molecular weight increased because degraded lignin became condensed, Mw = 12061 Da and PDI = 5.00.

### 2.5. Chemical Structure and Functional Groups of the Hydrotropic Lignins

**Figure 6:**
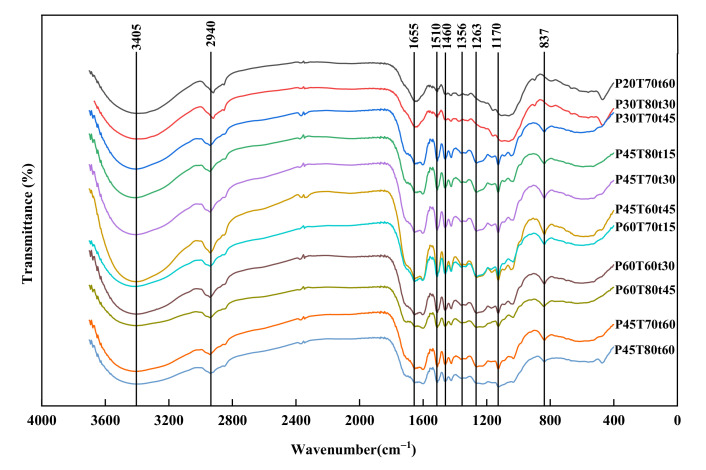
FT-IR spectra of lignin.

The FTIR spectrum of the hydrotropic lignins is illustrated in Figure 6. The absorption peak positions of the main functional groups of different hydrotropic lignin did not change. The stretching vibration peak of -OH in the cellulose, hemicellulose, and lignin was at 3405 cm^−1^; it was a broad peak for lignin, and the absorption of lignins of high CDF (>4000) has low intensity, which means the hydroxyl groups decreased. At 2940 cm^−1^, there are CH stretching vibration absorption peaks of the methyl group, methylene group and methine group, and the absorption intensity varied a little as well [31,32]. At 1655 cm^−1^, there is the conjugated C=O stretching vibration absorption peak, where the absorption peak is reduced, indicating that the relative content of p-hydroxy phenylpropane units is low. The peaks at 1510 cm^−1^ and 1460 cm^−1^ are assigned to the vibration of aromatic ring stretching. At 1356 cm^−1^, there are the C-H bending vibration absorption peaks on the aromatic ring. Additionally, the absorption intensity of the two places does not change much. At 1263 cm^−1^, the C-O bond stretching vibration absorption peaks in the aromatic ring methoxy group are very close together and in superposition to each other to form a wide peak because lignin contains both aromatic ethers, fatty ethers, and C-O structures in the carboxyl group. The in-plane and out-of-plane deformation of the benzene ring C-H structure in the guaiacyl propane unit caused the absorption peaks of 1170 cm^−1^ and 873 cm^−1^, respectively.

**Figure 7:**
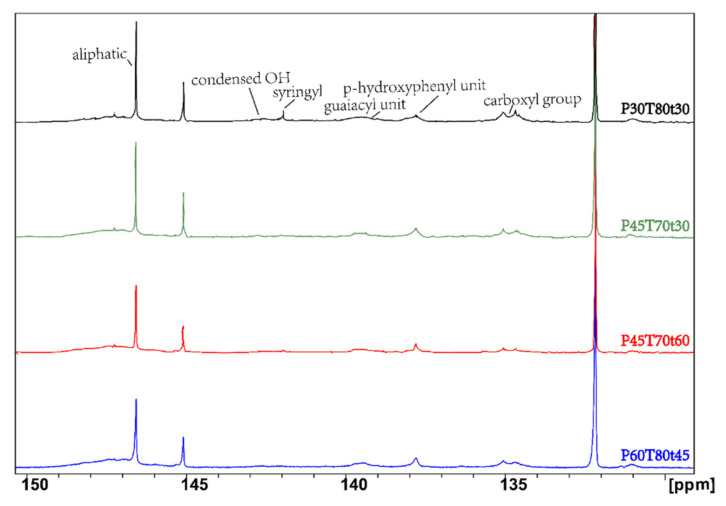
^31^P NMR spectroscopy of selected lignin samples in comparison. From top to bottom: P30T80t30, P45T70t30, P45T70t60, P60T80t45.

^31^P NMR spectroscopy is illustrated in Figure 7. Since the signal peaks of the corresponding lignin in different hydroxyl structures in the ^31^P NMR spectrum are well separated, and the chemical displacement is in significantly different regions, the characteristic functional groups such as phenolic hydroxyl, aliphatic hydroxyl, and carboxyl can be clearly distinguished in lignin. The positions of the absorption peaks of the lignin treated under different conditions are the same, but the absorption peak intensities are different. The signal peak of the aliphatic is 149.0–145.8 ppm, 144.8–143.1 ppm and 142.4–141.5 ppm are the signal peaks of condensed OH, 143.1–142.4 ppm is the signal peak of the syringyl unit (S), 140.0–138.8 ppm is the signal peak of the guaiacyl unit (G), 138.5–137.4 ppm is the signal peak of the p-hydroxyphenyl unit (H), and 136.0–133.8 ppm is the signal peak of the carboxyl group. According to the results of nuclear magnetic detection, the higher CDF, the higher the content of carboxyl and condensed OH, the higher the content of S-type and G-type lignin, the fewer aliphatic OH, and the smaller the change in H-type lignin.

The content of OH and COOH in lignin was calculated as listed in Table 3 (in mmol/g lignin). The decrease in aliphatic hydroxyl groups suggested that the aliphatic OH groups were oxidized and modified during the *p*-TsOH fractionation, such as the acid-catalyzed elimination reactions [26]. The high content of the phenolic hydroxyl implies the cleavage of β-O-4′ linkages [33]. P60T80t45′s CDF was higher than others, and it also had a higher amount of phenolic OH, suggesting that *p*-TsOH could break more β-O-4′ linkages and increase S and G phenolic hydroxyl groups at high CDF. Lignin esterification by severe conditions increased the carboxyl group content in hydrotropic lignin.

**Figure 8:**
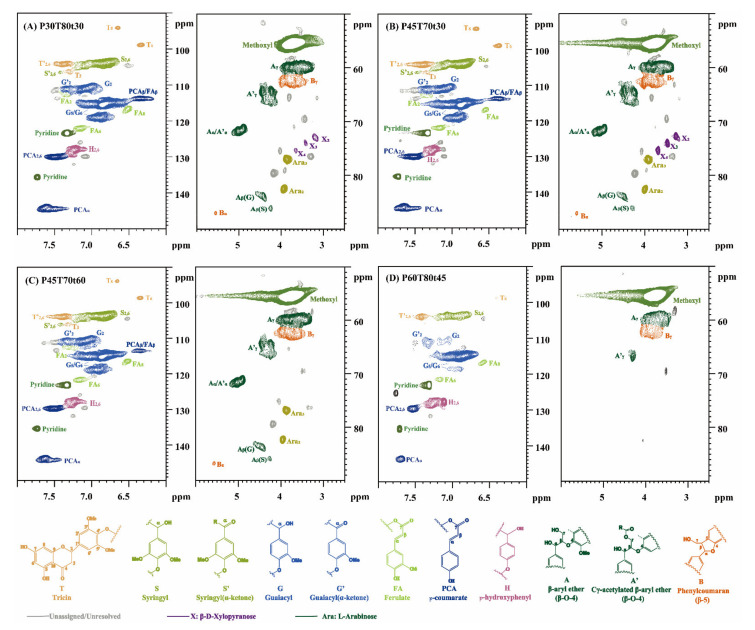
2D ^13^C-^1^H NMR HSQC spectra of selected lignin samples: (**A**) P30T80t30; (**B**) P45T70t30; (**C**) P45T70t60; (**D**) P60T80t45.

The main signals and substructures of lignin in the side chain (δC/δH 50–90/2.5–6.0) and the aromatic region (δC/δH 90–150/6.0–8.0) are shown in Figure 8. 2D ^13^C-^1^H HSQC NMR can determine specific carbon functionalities that cannot be identified using either ^13^C or ^1^H one dimensional spectra [34,35,36]. The assignments of main cross-signals of lignin and quantification of main chemical structures per 100 monomeric lignin units (100 Ar) are based on literature [36,37,38,39]. The quantification of main chemical structures per 100 monomeric lignin units (100Ar) and ratio, and linkages content was calculated as listed in Table 4. Integration of pCA _2/6_ was used for the quantification of pCA content; the integration of the FA_2_ crosspeak was used for quantitation of FA content.

The total aromatic areas are defined as:

I(C9) = 0.5 × I(S_2,6_) +0.5 I(S′_2,6_) +I(G_2_) +I(G′_2_) +0.5 ×I(H_2,6_). (3)

The content of internal linkages is calculated as:

β-O-4 = 100 × [I(β-O-4)]/[I(C9)]. (4)

β-β = 100 × [I(β-β)]/[I(C9)]. (5)

β-5 = 100 × [I(β-5)]/[I(C9)]. (6)

The content of aromatic units is relative (%):

S = 100 × 0.5 × [I(S_2,6_)]/[I(C9)]. (7)

S′ = 100 × 0.5 × [I(S′_2,6_)]/[I(C9)]. (8)

G = 100 × [I(G_2_)]/[I(C9)]. (9)

G′ = 100 × [I(G′_2_)]/[I(C9)] × 100. (10)

S: G = [(S + S′)]/[(G + G′)]. (11)

The side chain region of lignin mainly contains methoxyl (OMe), β–O–4′ linkages and carbohydrates, etc. OMe is accompanied by G or S structural units [41]. Correlation peaks from methoxyl groups and β–O–4′ linkages (A) were most prominent in the side chain region of all lignin preparations, followed by phenylcoumarans (B). Various signals associated with carbohydrates were also present, including β-D-xylopyranoside units (X) and arabinofuranoside units (Ara). The typical signals for major intercellular connections in the side chain region of lignin are mainly from the structure of β-O-4′, β-5′ and β-β′ [16,26]. The signals at δC/δH 71.5/5.00 ppm, 83.8/4.48 ppm, and 59.9/3.63 ppm are attributed to Aα, Aβ(G), and Aγ correlation in β-O-4′ substructures, respectively.

This indicated that temperature played a more critical role than acid concentration. For example, lignin from the two runs with moderate temperatures and fraction time: the β-O-4′ linkages content of P45T70t30, P30T80t30, and P45T70t60 varied little; it was around 30%. But P60T80t45 β-O-4′ linkage content was only 15.34%, approximately 50% of β-O-4′ linkages content was reduced compared to P30T80t30 lignin. This indicates that high temperature, elevated concentration, and extended fractionation duration play a critical role in eliminating β-O-4′ linkages to form condensed lignin. The pretreatment enhances the degradation of lignin as the severity increases. The β-O-4′linkages content of alkali oxygen lignin was only 7%, even lower than the P60T80t45 lignin [40].

The Bγ signal appeared in the side chain region, and the B linkage content was much lower than the A linkage, which may be because the B structure was more stable and difficult to extract by *p*-TsOH. The side chains of coumaric acid, ferulic acid, and tritice all contain an active olefin (Cα=Cβ) structure, and the olefin content reflects the reactivity of lignin to a certain extent. The absorption peak of side chain olefins was in the benzene ring region of lignin. The olefin content was expressed by the number of olefin carbon (VC) in 100 aromatic rings (100 Ar) [37]. The number of vinylic carbons was quantified using equation 12. The results (Table 5) showed that the VC/100 Ar content was 68.5(P30T80t30), 45.4 (P45T70t30), 35.0 (P45T70t60), and 21.1(P60T80t45), respectively. The VC/100 Ar content of milled wheat straw lignin was about 25 [42]. which indicated that the unsaturation in the side chain of hydrotropic lignin was high. The hydrotropic lignin had high reactivity. But the reactivity decreased with the increasing CDF.

VC/100Ar=2(I_FAα_ +I_pCAα_ + I_T3_)/[ I(C9) + I_FA2_ +( I_T’2,6_ + I_pCA2,6_)/2] × 100 (12)

The aromatic region of rice straw lignin was mainly composed of the basic structural units of H, G and S lignin. This indicates that rice straw cell walls were mainly enriched with G and S units along with a minor amount of hydroxyphenyl units. The signals that corresponded to p-coumarate (pCA) and ferulate (FA) structures were also observed clearly in the aromatic region. The G and S units were the main structures in rice straw. The signals derived from tricin (T) were also detected in the HSQC NMR aromatic region. Generally, the pretreatment efficiency is directly proportional to the amount of S units in the lignin [43]. The amount of H unit and G′ unit increased after high severity treatment, while the S unit decreased. The G units have a free C-5 position available for carbon-carbon bonds, which makes them relatively resistant to lignin depolymerization in pretreatment [44,45]. H units have a free C-3 and C-5 position available for carbon-carbon bonds. Moreover, the slight increase in the S/G ratio indicates that the G units were preferentially degraded. At low fractionation severities such as P30T80t30, P45T70t30, and P45T70t60, the S to G ratio and β-O-4 varied little, suggesting that the lignin structure was not significantly altered. The signal intensities of the pCA and FA were decreased with the increasing CDF. This suggests that the hydrotropic process could decompose the p-coumaric acid and ferulate structure.

### 2.6. Thermal Characteristics of Lignin

**Figure 9:**
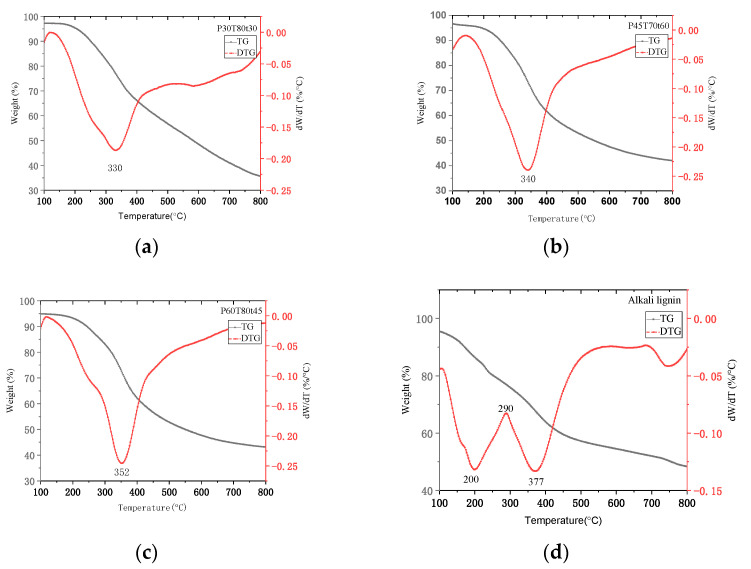
TG and DTG profile of selected lignin samples: (**a**) P30T80t30; (**b**)P45T70t60; (**c**) P60T80t45; (**d**) Alkali lignin.

Thermal characteristics of lignin can be obtained from thermos-gravimetry analysis (TGA) and differential scanning calorimeter (DSC). These analyses give useful data on specific temperatures where various heterogeneous reactions occur throughout the pyrolysis of biomass inside the TGA. TG and DTG profiles are shown in Figure 9. The thermal stability of lignin can be expressed by the maximum weight loss rate of lignin [31,32]. The maximum weight loss temperature of the hydrotropic lignin is basically in the range of 330–350 °C; this is due to the complex structure of lignin with phenolic hydroxyl and carbonyl groups and benzylic hydroxyl. The thermal degradation rate of the hydrotropic lignin showed a rapid upward trend in the range of 120–350 °C, and then gradually decreased as the temperature increased. The maximum weight loss temperature of P60T80T45 lignin was the highest at 352 °C. The maximum weight loss of the alkaline lignin had two peaks, the first thermal degradation rate was 0.13%·°C^−^^1^ at 200 °C, the second thermal degradation rate was 0.13%·°C^−1^ at 377 °C. These results indicated that the hydrotropic lignin is harder to be decomposed than the alkaline lignin.

**Figure 10:**
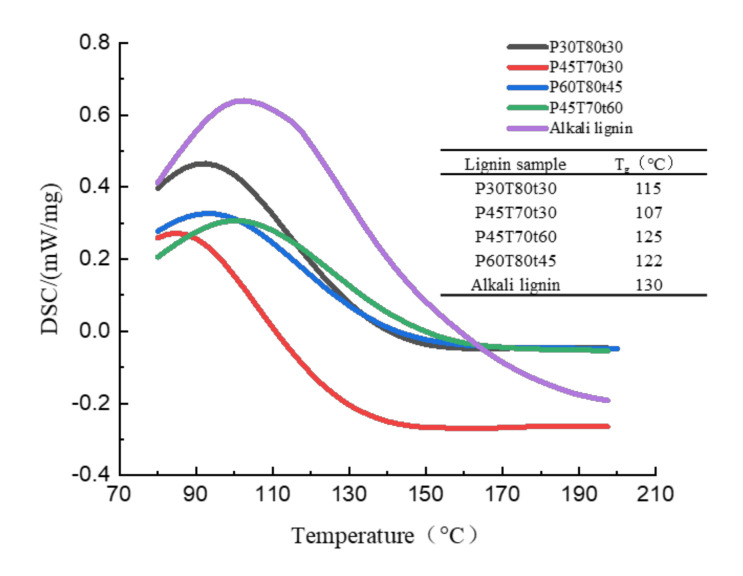
DSC profile and T_g_ of lignin samples.

The DSC curve can reflect the thermal effect of lignin. The glass conversion temperature (T_g_) of the hydrotropic lignin was in the range of 107–125 °C (Figure 10). T_g_ of the hydrotropic lignin increased a little with increasing severity. This may also indicate lignin condensation at high CDF. The glass transition temperature of alkaline lignin was 130 °C, higher than that of the hydrotropic lignin. Because the alkaline lignin was structurally modified under high temperature pretreatment, the low T_g_ of hydrotropic lignin, the low condensation degree, and higher activity was beneficial for its further catalytic or other valorization.

## 3. Materials and Methods

### 3.1. Chemicals

Rice straw was collected from Yancheng, Jiangsu Province, China. It was run through with a disk refiner, only one pass without any chemical treatment. The moisture of rice straw filament was approximately 10%. The *p*-TsOH (≥99.5%, AR) was purchased from Shanghai Macklin Biochemical co Ltd., Shanghai. China.

### 3.2. p-TsOH Fractionation and Alkali Fractionation of Rice Straw

*p*-TsOH fractionation was carried out according to the experimental flow schematics shown in Figure 1. For each fractionation, an acid solution to rice straw ratio of 10:1 was used (i.e., 20 g oven-dry (OD), and rice straw was fractionated in 200 g of the *p*-TsOH solution [21]). Fractionation runs were conducted in a 500 mL flask heated to the desired fractionation temperature by water bath. At the end of the preset time, 200 mL DI water was added to dilute the acid concentration to terminate the fraction. The solids and spent liquor were separated using vacuum filtration with the Whatman filer paper. Spent liquor was diluted to 2% acid concentration with water to precipitate the hydrotropic lignin. After settling for 24 h, the supernatant was decanted and the precipitate lignin was collected. To remove the remaining *p*-TsOH, the collected lignin was dialyzed using DI water until conductance reached 1 μS/cm as measured by a conductance meter (DDS-307, Shanghai INESA Scientific Instrument Co.,LTD, Shanghai. China). The lignin was then freeze-dried for further use.

Alkali fractionation: The rice straw was cooked using 10% NaOH in an oil bath rotary digester with a material to liquid ratio of 1:4. Impregnation and mixing were performed for 15 min, and rice straw was then cooked at 150 °C for 2 h, which included 1.5 h required to raise the temperature of the digester from room temperature to the cooking temperature of 150 °C. The cooked pulp and black liquor were separated with a 200 mesh cloth bag, and the pulp was washed thoroughly. Lignin in black liquor was precipitated by adjusting pH to 2 with 0.5 mol/L HCl and stirring for 1 h at room temperature. The crude lignin dissolved in 90% (*w*/*w*) acetic acid, and the soluble fraction was slowly introduced into deionized water. The precipitate was washed with deionized water thoroughly, and then was freeze-dried to obtain AL [40].

### 3.3. Atomic Force Microscopy (AFM) Analysis

A 0.1% lignin suspension was sonicated for 30 min. A drop of the suspension was dispersed on a mica substrate, air-drying at ambient temperature [20]. Atomic Force Microscopy (AFM, Bio MFP-3D, Asylum Research, USA) images of the air-dried lignin were taken in vibrating tapping mode using the manufacturer’s protocol. The height distributions of lignin from the AFM-measured topography of the sample were analyzed using the commercial software.

### 3.4. Particle Size Measurement

Lignin particle size distribution was measured at photon number 40–60 K with a nanometer laser particle size analyzer (BT-90, Bettersize Instruments Ltd., Dandong China).

### 3.5. Gel Permeation Chromatographic (GPC) Analysis

The lignin samples were acetylated before GPC analysis. In brief, 0.1 g of lignin was dissolved in 2 mL of mixed solution (pyridine and acetic anhydride *v*:*v*, 1:1), and stored for 72 h in dark. The solution was then transferred to 120 mL of ice water (containing 1 mL HCL) with a pipette. The precipitated lignin acetate was separated by centrifugation and washed twice with DI water, discarding all supernatants, and then air dried at room temperature for 48 h and then in a vacuum oven at 50 °C for an additional 24 h [21].

The molecular weights of the acetylated lignin samples were analyzed by GPC (LC-20 A, Shimadzu Co., Kyoto, Japan) with a RID to get weight-average (Mw) and number-weight (Mn) molecular weights and polydispersity index (PI, Mw/Mn). Lignin acetate (2 mg) was dissolved in 1 mL of tetrahydrofuran (THF). The KF-803 gel column (300 mm × 8.0 mm) was calibrated with polystyrene standards (peak average molecular weights of 43,600, 30,000, 20,000, 10,000, 4050, 2400 Da). THF was used as an eluent at a flow rate of 1 mL/min. The column temperature was 35 °C.

### 3.6. Fourier Transform-Infrared (FT-IR) Spectroscopy Analysis

FT-IR (FTIR-650, Tianjin GangDong Sci&Tech Development Co., Ltd, Tianjin, China) was used to analyze the changes of lignin chemical bonds and functional groups after pretreatment. All samples were pressed by KBr prior to testing.

### 3.7. Nuclear Magnetic Resonance (NMR) Spectroscopic Analyses of Lignin

^31^P NMR and 2D ^1^H-^13^C heterogeneous single quantum correlation (HSQC) NMR of lignin samples were analyzed using a Bruker AVIII 600 MHz (Switzerland) spectrometer. For 31P NMR analysis, 25 mg of dried lignin was dissolved in 400 μL of anhydrous pyridine-d*5*/CDCl_3_ (1.6/1, *v*/*v*) solution. Then, 150 μL of cyclohexanol (4.0 mg/mL, internal standard) and chromium (III) acetylacetonate (3.6 mg/mL, relaxation regent), prepared using anhydrous pyridine-d*5*/CDCl_3_ solution, were mixed well with lignin solution and then added to a 75 μL phosphitylating regent (2-chloro-4,4,5,5-tetramethyl-1,2,3-dioxaphospholane, TMDP), which was shaken at room temperature for 1 min, and then analyzed immediately [26,46]. The PULCON (pulse length-based concentration determination) method was applied to the phosphorylated lignin sample to collect 250 scans, and the spectrum was calibrated according to phosphorylation agent at 145.1 ppm.

For 2D-HSQC NMR experiments, 60 mg of lignin were dissolved in 0.6 mL of dimethyl sulfoxide-d_6_/pyridine-d_5_ (4:1) [21]. HSQC experiments were performed using the Bruker standard pulse program hsqcetgisisp2.2. Spectra were acquired using 40 scans, an interscan delay of 1 s, a 12-ppm sweep width in F2 (1 H), 1024 data points for an acquisition time of 85 ms and a 215-ppm sweep width in F1 (13C), and 512 increments with 50 % nonuniform sampling density. Topspin 4.0.9 was used for interactive integration of the cross peaks after the central DMSO solvent peak was referenced at *δ* 13C, 39.5 ppm; *δ* 1 H, 2. 5 ppm.

### 3.8. Thermogravimetric (TG) Analysis

Thermogravimetric studies were performed using a NETZSCH TG 209 F1 thermal analyzer made by the Shimadzu Company, Kyoto, Japan. The thermal decomposition characteristics of the lignin samples were determined with an HTG-3 thermal analyzer (TGA209 F1, NETZSCH Scientific Instruments Trading Ltd., Selb, Germany). A total of 5–10 mg of the samples were placed in an alumina crucible and heated from 30 to 800 °C, at heating rates of 10 °C/min. Nitrogen was used as an inert gas with a flow rate of 50 mL/min [47,48].

Thermogravimetric studies were performed using a NETZSCH TG 209 F1 thermal analyzer made by the Shimadzu Company, Kyoto, Japan. The thermal decomposition characteristics of the lignin samples were determined with an HTG-3 thermal analyzer (TGA209 F1, NETZSCH Scientific Instruments Trading Ltd., Selb, Germany). A total of 5–10 mg of the samples were placed in an alumina crucible and heated from 30 to 800 °C, at heating rates of 10 °C/min. Nitrogen was used as an inert gas with a flow rate of 50 mL/min [47,48].

### 3.9. Differential Scanning Calorimetric (DSC) Analyses

The lignin glass transition temperature T_g_ was obtained through DSC analyses using a calorimetric system (DSC-0306, NETZSCH Scientific Instruments Trading Ltd., Selb, Germany). Lignin of 5–10 mg ran at a heating rate of 10 °C/min between 40 °C and 200 °C, under nitrogen flow (40 mL/min) [21].

## 4. Conclusions

This study reported that the rice straw hydrotropic lignin was successfully isolated from *p*-TsOH fractionation, then characterized comprehensively. The value of CDF indicated the fractionation severity. The hydrotropic rice straw lignin particle was in nanoscale. The PDI of hydrotropic lignin was higher than that of alkaline lignin. Compared with alkaline lignin, especially at low fractionation severities, the hydrotropic lignin has well-preserved β-aryl linkage content, high reactivity, large molecular weight and low glass transition temperature, excellent for direct application in composites or catalytic valorization.

## Figures and Tables

**Table 1:** Rice straw lignin removal rate under a range of *p*-TsOH fraction conditions.

Sample	Concentration (%)	Temperature (°C)	Time (min)	Lignin Removal Rate (%)	CDF (min·mol/L)
P20T70t60	20	70	60	18.3	1134.7
P45T60t45	45	60	45	31.4	1593.5
P30T70t45	30	70	45	32.8	1638.9
P60T60t30	60	60	30	37.5	2172.9
P30T80t30	30	80	30	39.9	2396.0
P45T70t30	45	70	30	40.2	2442.1
P60T70t15	60	70	15	43.0	2497.6
P45T80t15	45	80	15	43.1	2677.7
P45T70t60	45	70	60	46.5	4884.3
P45T80t60	45	80	60	50.4	10,710.8
P60T80t45	60	80	45	52.4	16,430.9

**Table 2:** Mw, Mn and PDI of hydrotropic lignin and the alkaline lignin.

Sample	Mw/Da	Mn/Da	PDI
P30T80t30	16055	2876	5.58
P30T70t45	13394	2671	5.01
P45T80t15	10537	2576	4.09
P45T70t30	11074	2587	4.28
P45T60t45	14276	2882	4.95
P60T70t15	11458	2550	4.49
P60T60t30	10111	2593	3.90
P60T80t45	12061	2413	5.00
Alkaline lignin	4397	1428	3.08

**Table 3:** Quantification of the functional groups in lignin by ^31^P NMR spectroscopy.

Sample	CDF (min·mol/L)	Aliphatic OH	Phenolic OH (mmol/g)				COOH
			Syringyl (S)	Guaiacyl (G)	*p*-Hydroxyl (H)	Condensed	
P30T80t30	2396.0	2.51	0.13	0.44	0.40	0.31	0.69
P45T70t30	2442.1	2.67	0.14	0.47	0.44	0.32	0.52
P45T70t60	4884.3	2.16	0.13	0.42	0.35	0.29	0.69
P60T80t45	16,430.9	1.73	0.21	0.50	0.40	0.50	0.88

**Table 4:** Semi-quantitative analysis of lignin units and ratio, and linkages based on 2D-HSQC spectra.

Sample	S	S′	G	G′	H	FA	pCA	A	B	S/G
P30T80t30	26.93	3.63	44.29	13.73	11.43	3.50	16.92	31.44	1.51	0.53
P45T70t30	30.56	1.59	36.09	19.06	12.70	2.98	19.83	33.97.	2.21	0.58
P45T70t60	32.96	0.83	34.97	18.67	12.58	2.58	14.65	29.81	2.17	0.63
P60T80t45	32.53	0.00	26.28	8.24	32.95	0.64	10.94	15.34	0.00	0.94
Milled wood lignin [40]	26	NA	65	NA	9	4	32	56	2	0.4
Alkali oxygen lignin [40]	23	NA	50	NA	27	4	1	7	1	0.5

**Table 5:** The number of olefin carbon (VC) calculation.

Sample	I(C9)	I_FAα+_ I_pCAα_	I_T3_	I_T’2,6_	I_FA2_	I_pCA2,6_	VC/100Ar
P30T80t30	0.113	0.0308	0.0165	0.0046	0.004	0.0382	68.4
P45T70t30	0.104	0.0277	0.0031	0.0162	0.0031	0.0412	45.4
P45T70t60	0.097	0.0201	0.0012	0.0158	0.0025	0.0284	35
P60T80t45	0.07	0.0091	0	0.0154	0.0009	0.0154	21.1

## Data Availability

The data presented in this study are available on request from the corresponding author.

## Sample Availability

Samples of the compounds are not available from the authors.
